# Brain abscess formation as a CSF shunt complication: a case report

**DOI:** 10.1186/1757-1626-2-110

**Published:** 2009-01-31

**Authors:** Aimun AB Jamjoom, Abrar R Waliuddin, Abdulhakim B Jamjoom

**Affiliations:** 1 Section of Neurosurgery, King Khalid National Guards Hospital, PO Box 9515, Jeddah 21423, Saudi Arabia

## Abstract

The formation of a brain abscess as a result of a cerebrospinal fluid shunt complication is extremely rare in the literature with only 7 cases reported in the last 20 years. We report a patient that developed a brain abscess adjacent to a functioning ventricular catheter in the presence of shunt infection by another pathogen. Clinicians should consider this complication in any shunted patient with clinical features of infection and suggestive changes on imaging however subtle. Expedited standard management for the abscess and the CSF shunt infection, if present, should be employed. Removal of all non-functioning catheters should be encouraged.

## Introduction

The insertion of a cerebrospinal fluid (CSF) shunt remains the most common operation for the treatment of hydrocephalus. These devices are associated with a number of varied and well-documented complications. Shunt infection is an important and distressing complication that occurs in 5–10% of patients.[1,2] It usually presents with shunt failure but in severe cases can progress to ventriculitis.[1,2] Brain abscess on the other hand is a well-recognized lesion that is reported to occur in 3.5-cases/500,000 population/year.[3] It commonly develops as a result of hematogenous spread of organisms from a distant focus, contiguous spread, operative procedures and penetrating cranial trauma. The formation of a brain abscess as a result of a shunt complication is extremely rare in the literature with only 7 cases reported in the last 20 years and another 4 cases reported earlier to that. [4-10] In this article we report a patient that developed a brain abscess adjacent to a functioning ventricular catheter in the presence of CSF shunt infection by another pathogen. It is hoped that presentation of the case will not only draw clinicians' attentions to this rare and important shunt complication but also allow us to make some observations about the clinical characteristics of brain abscess in shunted patients in general.

## Case history

A 9-month-old male child with congenital hydrocephalus was admitted to our neurosurgical unit and underwent the insertion of a right parietal ventriculo-peritoneal (VP) shunt. He was known to have multiple congenital anomalies that had been detected from birth. These included a unilateral cleft lip and palate and a left sided inguinal hernia for which he had surgery. In addition he had bilateral peri-auricular tags, small membranous muscular ventricular septal defect (VSD), fused upper ribs and hypospadias. The patient tolerated the shunting procedure well and apart from delays in his developmental milestones, he remained well at follow up.

**Figure 1:**
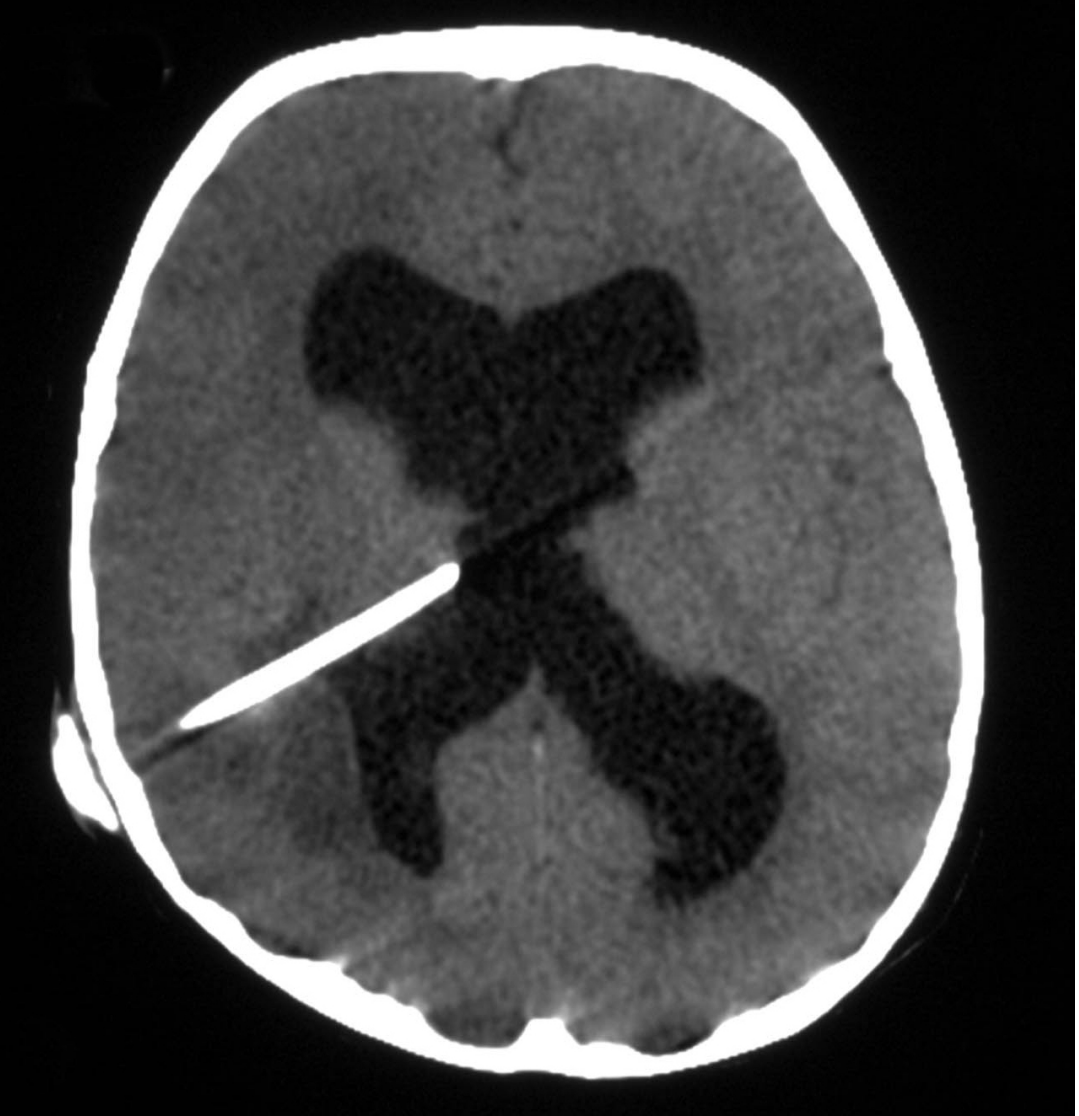
**Brain CT (Plain): **showing dilated ventricles and right parietal low-density area adjacent to the ventricular catheter and causing slight indentation in the occipital horn.

**Figure 2:**
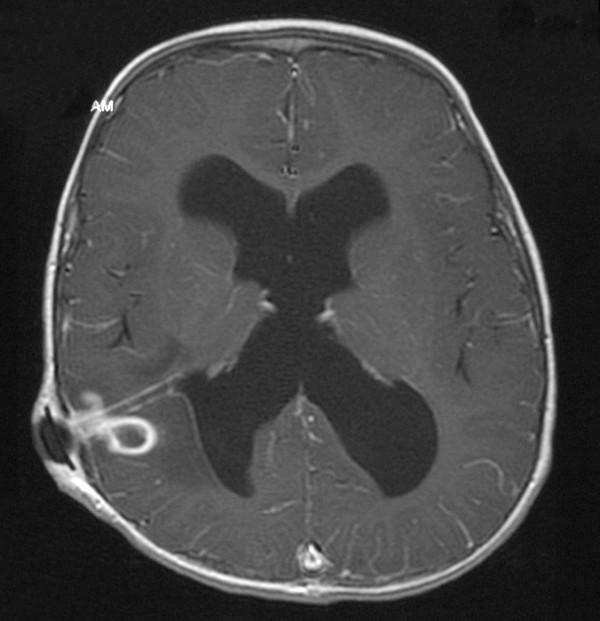
**MRI (T1 + contrast): **showing a small ring-enhancing lesion with mild surrounding edema adjacent to the ventricular catheter and ventricular dilatation.

At 2 years of age, he was readmitted with a few days history of low-grade fever, vomiting, lethargy and one generalized convulsion. Plain brain CT (Figure 1) showed dilated ventricles and a right parietal low-density area adjacent to the ventricular catheter causing slight indentation in the occipital horn. Contrast MRI (Figure 2) showed a small right parietal ring-enhancing lesion adjacent to the ventricular catheter with surrounding edema causing mild mass effect. There was evidence of ventricular dilatation even though the valve was functioning well clinically. A CSF sample from the shunt system grew Staphylococcus aureus. The patient underwent removal of the VP shunt and insertion of a frontal external ventricular drain (EVD). In addition, 3 mls of pus were aspirated from the brain abscess. The EVD was converted to a VP shunt 10 days later after obtaining three consecutive negative CSF cultures. The aspirated pus grew Enterobacter cloacae and Bacteroides. Abdominal CT scan, which was done, to look for intra-abdominal pathology was normal. The patient was initially managed by intravenous Ceftriaxone, Vancomycin and Flagyl, which based on sensitivity results were later changed to Cloxacillin, Meropenem and Flagyl. The intravenous antibiotics were prescribed for 14 days and were followed by oral Ciprofloxacin and Flagyl for another three weeks. The patient made a good recovery and a follow up CT scan four weeks later confirmed complete resolution of the brain abscess.

## Discussion

The formation of a brain abscess in a shunted patient can be shunt related or unrelated. A shunt related abscess is either intraparenchymal adjacent to the ventricular catheter as in our case [4-10] or intraventricular, which is also referred to as "loculated empyema", or "pyocephalus".[1,2,11] On the other hand an intraparenchymal abscess that is located at a distance from the ventricular catheter is likely to be shunt unrelated.[12] Review of the 7 cases reported over the last two decades revealed that the abscess can develop from one month to 6 years (median 1 year) after the shunt placement. [4-10] In addition, the abscess can be found adjacent to a functioning [4-6,9] or a non-functioning ventricular catheter.[7,8,10] The latter finding has been used to support the argument that all non-functioning catheters need to be removed. Naturally the remote risk of latent abscess formation if the catheter is left behind must be weighed against the risk of bleeding during the removal of an adherent catheter.[7,8,10]

The brain abscess in our patient grew Enterobacter cloacae and Bacteroides. A variety of organisms have been identified as the pathogens in the reported cases. These include: Escherichia coli,[4,10] Enterobacter Cloacae,[4] Enterococcus avium,[9] Klebsiella oxytoca,[5] Proteus mirabillis, [8] Staphylococus aureus [6] and Propionibacterium acnes.[7] Abscesses caused by enteric organisms have been attributed to an ascending infection from bowel perforation. The latter was diagnosed in two patients by evidence of staining of the distal part of the peritoneal catheter by faecal matter or bile-salt.[4,5] However, ascending infection can occur in the absence of evident bowel perforation as in our case.[9] In addition, the abscess can be attributed to microbial colonization of the external surface of the shunt from contamination during the initial surgery, [7] from an infective process close to the shunt system, from shunt exposure due to wound dehiscence [6] or from multiple valve puncturing for CSF samples.[9] Interestingly, 4 of the 7 reported patients had tumour related hydrocephalus [6-9], which suggests that the presumed immunity impairment in brain tumour patients taking steroids may be a predisposing factor. The congenital heart disease (VSD) in our patient may have been relevant as such patients are at risk of developing brain abscess in general.

Our patient had shunt CSF infection that was caused by a different pathogen from the abscess. Shunt CSF infection at the time of diagnosis of the abscess was observed in 4 of the 7 reported cases.[4-6,9] In addition, in two of these patients, [5,9] the pathogens cultured from the CSF were different from those in the abscess. Such finding is not surprising and can be explained by the variation in bacteria's adhesiveness and motility against the CSF flow.[4,9] Patients with a shunt related brain abscess require removal of the shunt, EVD, aspiration of the abscess and intravenous antibiotics. In addition, the patient should be investigated for the primary source of the infection particularly if ascending infection is suspected. The EVD can be changed to a shunt in a new location when the CSF is sterile on repeated cultures and the abscess appears resolved on CT scanning.

A patient with a severe CSF shunt infection is at risk of developing ventriculitis, multiloculated hydrocephalus and loculated ventricular abscess/empyema (pyocephalus).[1,2,11,13] The infection is usually caused by organisms similar to those responsible for the CSF shunt infection with gram-negative organisms being the most frequently implicated.[2] Treatment is usually by removal of the shunt, aspiration of the ventricular loculated abscess/empyema, EVD and intravenous antibiotics. The aspiration procedure can be refined by using navigation or stereotactically guided endoscopic technique [11] and in severe infection intraventricular antimicrobial therapy may be required.[13] Once the CSF is sterile and the abscess has resolved endoscopic fenestration of the cysts with a single shunt or insertion of multiple shunts can be considered.[2]

A shunted patient can develop an intraparenchymal brain abscess that is unrelated to the shunt in its origin. Such an abscess will be distant from the ventricular catheter and is usually caused by organisms similar to the primary source sepsis.[12] A shunt CSF sample should be cultured and in the absence of evidence of CSF infection, the abscess can be treated independently by aspiration and intravenous antibiotics while the shunt system should be carefully monitored for potential infection.

## Conclusion

Brain abscess formation as a CSF shunt complication is rare with only few cases in the literature. Clinicians should consider this complication in any shunted patient with clinical features of infection and suggestive changes on the plain CT scan however subtle. A contrast MRI or CT will be required to demonstrate the pathology. The location of the abscess on the MRI, the causative pathogen and the presence or absence of concomitant shunt CSF infection are useful in providing clues towards the pathogenesis of the abscess. Expedited standard management for the abscess and the CSF shunt infection should be employed. Removal of all non-functioning catheters should be encouraged.

## Consent

Written informed consent was obtained from the father of the patient for publication of this case report and accompanying images. A copy of the written consent is available for review by the Editor-in-Chief of this journal.

## Abbreviations

CSF: cerebrospinal fluid; VP: ventriculo-peritoneal; CT: computed tomography; MRI: magnetic resonance imaging; EVD: external ventricular drain.

## Competing interests

The authors declare that they have no competing interests.

## Authors' contributions

AABJ prepared the manuscript, references and literature search. ARW prepared the images and assisted AABJ in preparing the references and discussion. ABJ was the operating surgeon and was responsible for the inception of the paper, critical review and the final revision of the paper. All authors read ad approved the final manuscript
